# Evaluation of right ventricular myocardial deformation properties in fetal hypoplastic left heart by two-dimensional speckle tracking echocardiography

**DOI:** 10.1007/s00404-022-06857-x

**Published:** 2023-02-10

**Authors:** Christian Enzensberger, Oliver Graupner, Stefanie Fischer, Markus Meister, Maleen Reitz, Malena Götte, Vera Müller, Aline Wolter, Johannes Herrmann, Roland Axt-Fliedner

**Affiliations:** 1 Department of Obstetrics and Gynecology, University Hospital Aachen, RWTH University, Pauwelsstraße 30, 52074 Aachen, Germany; 2 Departement of Obstetrics and Gynecology, University Hospital Rechts der Isar, Technical University, Munich, Germany; 3 Division of Prenatal Medicine, Departement of Obstetrics and Gynecology, Justus Liebig University, Giessen, Germany; 4 Statistical Consulting Service Giessen, Giessen, Germany

**Keywords:** Fetal hypoplastic left heart syndrome, Fetal cardiac function, Speckle tracking echocardiography, Left ventricular endocardial fibroelastosis

## Abstract

**Purpose:**

Right ventricular (RV) function influences the outcome of hypoplastic left heart (HLH) patients. This study aimed to confirm the assumption of prenatal RV remodeling and possible influencing factors of myocardial restructuring using two-dimensional speckle tracking echocardiography (2D STE).

**Methods:**

This is a retrospective cross-sectional cohort study including HLH fetuses and gestational age-matched controls. Based on a four-chamber view, cine loops were stored with 60 frames per second. Global longitudinal peak systolic strain (GLPSS) of the RV was retrospectively determined and compared to healthy controls. Furthermore, HLH subgroups were built according to the presence of left ventricular endocardial fibroelastosis (LV-EFE) and restrictive foramen ovale (FO) to investigate the effect of these compromising factors on myocardial deformation.

**Results:**

A total of 41 HLH fetuses and 101 controls were included. Gestational age at fetal assessment was similarly distributed in both groups (controls: 26.0 ± 5.6 weeks vs. HLH: 29.1 ± 5.6 weeks). Relating to RV-GLPSS values, fetuses with HLH demonstrated lower mean values than healthy control fetuses (− 15.65% vs. − 16.80%, *p* = 0.065). Cases with LV-EFE (*n* = 11) showed significantly lower mean values compared to such without LV-EFE (*n* = 30) (RV-GLPSS: − 12.12% vs. − 16.52%, *p* = 0.003). No significant differences were observed for cases with FO restriction (*n* = 10).

**Conclusions:**

In HLH the RV undergoes prenatal remodeling, leading to an adaptation of myocardial function to LV conditions. Further explorations by STE should expand knowledge about RV contraction properties in HLH and its impact on surgical outcome.

**Supplementary Information:**

The online version contains supplementary material available at 10.1007/s00404-022-06857-x.

## What does this study add to the clinical work?

**Table:** 

In Hypoplastic Left Heart the right ventricle undergoes prenatal remodeling reflected by different myocardial deformation properties. This can be interpreted as an adaptation of myocardial function to left ventricular conditions.	

## Introduction

Hypoplastic left heart (HLH) is one of the most severe forms of cardiac abnormalities detectable during gestation by fetal echocardiography [1]. The incidence of HLH is estimated at 0.16–0.36 per 1000 live births, accounting for approximately 4.8–9% of all congenital heart diseases (CHD) [2–4]. HLH contains four main subgroups attributed to anatomic combinations of valvular dysgenesis, namely stenosis or atresia of the mitral and/or aortic valve: mitral atresia/aortic atresia (MA/AA), mitral stenosis/aortic stenosis (MS/AS), mitral stenosis/aortic atresia (MS/AA) and mitral atresia/aortic stenosis (MA/AS) in association with a ventricular septal defect leading to a hypoplasia of the left ventricle (LV) and the ascending aorta [5].

Advanced ultrasound techniques for the evaluation of fetal cardiac function could supply valuable predictive information about postnatal conditions, especially in HLH cases, in which cardiac output depends on the right ventricle (RV) [6, 7]. The fact that altered RV filling patterns occur in HLH has already been described [8, 9]. Two-dimensional Speckle Tracking (2D STE) is a technique for the evaluation of myocardial deformation. It has shown good reproducibility and feasibility in several studies [10–12]. It is assumed that 2D STE is an angle-independent technique. However, recent data shows that there may actually be differences in STE data obtained depending on fetal heart position [13].

The assessment of cardiac function, especially of the RV, is increasingly becoming the focus of interest in HLH fetuses [8, 9, 14–18]. Prenatal changes in RV function may be crucial for the future function of the single ventricle, as postnatal RV dysfunction is an important risk factor for the survival of HLH children in the course of multistage surgery [5, 18–20]. Long-term results of surgical palliation and Hybrid procedure in HLH patients reveal poor survival rates within the subgroup of MS/AA especially those presenting with LV endocardial fibroelastosis (EFE) [5, 19, 20]. EFE is defined as an endocardial thickening based on an increased amount of collagen and elastin fibers. It might be present in fetuses with CHD and is mostly detected simultaneously with the incidence of aortic valve stenosis [21–23]. There have been discussions as to whether EFE could be the cause of relative diastolic and systolic dysfunction in fetuses with left heart disease due to its inelastic fibrotic properties [8, 24, 25].

In this study, we used 2D STE, first, for evaluation of RV myocardial function in HLH fetuses and, secondly, to test for possible influences of LV-EFE and foramen ovale (FO) restriction on RV function. We hypothesized that changes in myocardial deformation of the RV in HLH fetuses would be detectable during gestation.

## Methods

### Study population

This is a retrospective study including pregnant women who were referred for fetal echocardiography to the Department of Fetal Diagnosis and Therapy at the University Hospital Giessen and Marburg from August 2012 to March 2018. Institutional review board approval was given (Protocol No. 209/11). Inclusion and exclusion criteria for healthy control fetuses were as previously described [9]. Inclusion criteria for the HLH group were the following:

- Fetuses with severe MS or MA and/or severe AS or AA
- Fetuses with borderline left ventricle (BLV) defined as a small—diminutive left ventricle (confirmed by the measurement of fetal LV *z*-scores [26]) with intrinsically open valves (MS and/or AS) [9].

In contrast to our previous work, we also included cases with restrictive foramen ovale (FO). The evidence of atrial restriction or premature closure of FO was diagnosed by dilated pulmonary veins or pulsatile flow in the pulmonary veins by spectral Doppler [14, 20]. We decided to investigate cases with restrictive FO both in the overall HLH collective and in a generated subgroup analysis (with and without restrictive FO). Due to the assumed functional impairment of the RV in the case of restrictive FO, we suspected this condition to influence the myocardial deformation properties of RV significantly. Fetuses with further intracardiac abnormalities as well as structural or chromosomal anomalies were excluded. Furthermore, maternal conditions with possible hemodynamical effects, such as diabetes or preeclampsia acted as exclusion criteria as previously described [9].

According to the mentioned criteria above, two main groups were formed: the control group and the HLH group. For subgroup analysis HLH study population was first divided into cases with, and without LV-EFE. Second, HLH cases were divided in those with patent FO and those with restrictive FO. To support our hypothesis more convincingly we also analyzed our data without BLV diagnosis.

### Echocardiography

In every patient, a complete fetal echocardiography scan was performed in a standardized manner with transverse and longitudinal planes by experienced operators on either a Toshiba Artida, an Aplio 500 or an Aplio i900 system (Toshiba Medical Systems Corporation, Otawara, Tochigi, Japan). To obtain video loops of a high-resolution, zoomed B-Mode of an apical or basal four-chamber view (4CV), a 1–5-MHz curved array probe (PVT 375 BT; Toshiba Medical Systems Corporation) was used. Based on previous studies the B-mode image depth was reduced and the sector width was narrowed [10]. To ensure high image quality, attention was taken to a clearly delineation of the RV and LV free wall as well as of the interventricular septum. To achieve better results, it was also intended to obtain recordings in the absence of fetal movements. The cine loops were digitally stored in Digital Imaging and Communications in Medicine (DICOM) format with 60 frames per second.

### 2D speckle tracking echocardiography

Speckle tracking is an application of 2D-CPA technology to ultrasound cine data. Based on post-processing 2D image frame-by-frame analysis, movement of the entire myocardium can be investigated. This method does not make use of Doppler information, so there is no Doppler angle dependency. Offline analysis was performed on an external workstation equipped with the Image Arena software (TomTec Imaging Systems, Image-Arena version 4.6, Unterschleissheim, Germany). Apical or basal four-chamber view (4CV) of good quality 2D B-mode cine loops, namely with a maximum expansion of the RV and a well-visible valve plane, was chosen for STE analysis [27].

Various cardiac cycles of one patient were identified by anatomical M-Mode to select one in which automatic tracking of the endocardial border functioned well. Heart cycles in which segments could not be tracked properly have not been used for analysis. After one fetal heart cycle was identified and selected by anatomical M-mode, we used the closure of the mitral and tricuspidal valve as orientation for the determination of the end systole.

Fetal heart rate was calculated on the basis of a heart cycle duration. Afterward strain measurement of the RV was performed. In a 3-point-analysis (septal annulus, lateral annulus, apex) endocardial borders of every cardiac cavity were defined (endocardial tracing). This methodology of offline analysis was previously described for the assessment of myocardial deformation properties in fetal humans [27, 28] and lamb hearts [29].

Global right ventricular longitudinal peak systolic strain (RV GLPSS) and strain rate (RV LSR) are displayed graphically and numerically as the calculated mean values for each of the three individual segments (RV free wall: basal, middle, apical; RV septal: basal, middle, apical). The software then detects the ventricular contour and the operator is able to assess the tracking quality. If tracking seemed inadequate, the operator was able to adjust the three points. Insufficient strain analyses due to poor quality of 4CV (e.g. lack of a clear endocardial line) were excluded from the study.

Intraobserver and interobserver variability of 2D-STE measurements were assessed in a subset of 20 echocardiograms from randomly selected control and HLH fetuses at various gestational ages. Two operators analyzed the same images independently.

### Statistical analysis

The data analysis was realized with IBM Statistics (Version 25). All group comparisons are analyzed in ANCOVA models with gestational age as a covariate. Dependent variables are GLPSS and LSR. Four different grouping variables (1. All HLH cases vs. Controls, 2. HLH with LV EFE vs. HLH without LV EFE vs. Controls, 3. HLH with FO restriction vs. HLH without FO restriction vs. Controls, 4. All HLH cases without BLV cases vs. Controls) were tested for each dependent variable (RV GLPSS, RV LSR), so eight models were computed. The interaction gestational age*group was not informative in all models, so it was excluded for reasons of model parsimony. Heterogeneity of variances was accounted for using procedure MIXED where the heterogeneity could be modeled.

For the comparisons between groups, the adjusted mean values (margins) from the models are reported in the result section. Multiple pairwise comparisons between the groups were corrected for inflation of type 1 error, using the Bonferroni correction. Due to the very different sample sizes of the groups the *p*-values of paired comparisons should be interpreted carefully—it may be that in the case of small sample sizes even considerable differences cannot be confirmed statistically (“significant”).

For reproducibility analysis, interobserver reliability was analyzed between two raters for GLPSS and for LSR, as well as intraobserver reliability between two measurements of one rater for the same parameters. The intraclass correlation coefficient (ICC two-way random, absolute agreement, single rater) was used for interobserver as well as for intraobserver variability. Values of 0.7–0.8 for the intraclass correlation coefficient indicate good agreement and values > 0.8 strong agreement between measurements. Additionally, Bland Altman analyses (procedure concord, using Stata version 16.1) complement the reproducibility analysis and were conducted for the described analyses. All *p*-values were considered significantly different at *p* < 0.05.

## Results

### Baseline characteristics of the study cohort

41 fetuses with HLH and 101 healthy fetuses were enrolled for final analysis. Gestational age was similarly distributed in both groups (controls: 26.0 ± 5.6 weeks vs. HLH: 29.1 ± 5.6 weeks). In 31 out of 41 HLH fetuses left-to-right shunt via the FO and retrograde aortic arch flow from the ductus arteriosus was observed. In ten cases there was no patent FO but a restrictive ASD or evidence for premature closure of FO. In 11 cases LV-EFE was diagnosed. Baseline characteristics and postnatal treatment of the study cohort (according to the type of HLH and type of treatment) are described in Table 1.

**Table 1:** Demographic characteristics of 41 fetuses with hypoplastic left heart (HLH) that underwent Speckle Tracking analysis for evaluation of right ventricular function

Case	GA at examination (weeks)	Type of HLH	FO	GA at delivery (weeks)	Mode of delivery	Perinatal/postnatal treatment		
1	31 + 1	MA, AA	Patent	38 + 5	SVD	Compassionate care		
2	22 + 1	MA, AA	Patent	41 + 2	SVD	Compassionate care		
3	21 + 3	MA, AA	Restrictive	30 + 1	SVD	Compassionate care		
4	29 + 5	MA, AA	Patent	39 + 2	SVD	Hybrid proc. (PAB, PDA stenting)	HTX	
5	34 + 5	MA, AA	Patent	38 + 6	CS	Hybrid proc. (PAB, PDA stenting)	Ex	
6	31 + 6	MA, AA	Patent	38 + 5	SVD	Hybrid proc. (PAB, PDA stenting)	CS II	TCPC
7	33 + 5	MA, AA	Patent	38 + 1	SVD	Hybrid proc. (PAB, PDA stenting)	CS II	TCPC
8	28 + 0	MA, AA	Patent	38 + 4	CS	Hybrid proc. (PAB, PDA stenting)	CS II	TCPC
9	36 + 0	MA, AA	Restrictive	37 + 6	CS	Hybrid proc. (PAB, PDA stenting)	CS II	TCPC
10	26 + 5	MA, AA	Restrictive	40 + 0	SVD	Hybrid proc. (PAB, PDA stenting)	CS II	TCPC
11	26 + 6	MA, AA, EFE	Restrictive	40 + 1	SVD	Hybrid proc. (PAB, PDA stenting)	CS II	TCPC
12	24 + 0	MA, AA	Patent	TOP				
13	37 + 5	MA, AS	Patent	38 + 3	SVD	Hybrid proc. (PAB, PDA stenting)	CS II	TCPC
14	19 + 6	MS, AA	Patent	39 + 1	CS	Hybrid proc. (PAB, PDA stenting)	HTX	
15	22 + 2	MS, AA	Patent	39 + 3	SVD	Hybrid proc. (PAB, PDA stenting)	CS II	Ex
16	32 + 5	MS, AA	Patent	39 + 5	SVD	Hybrid proc. (PAB, PDA stenting)	CS II	
17	28 + 1	MS, AA, VSD	Patent	38 + 4	SVD	Hybrid proc. (PAB, PDA stenting)	CS II	TCPC
18	25 + 2	MS, AA, EFE	Restrictive	38 + 1	SVD	Hybrid proc. (PAB, PDA stenting)	CS II	TCPC
19	28 + 3	MS, AA, EFE	Patent	40 + 0	CS	Hospital transfer (Norwood op)		
20	28 + 1	MS, AA	Restrictive	37 + 3	CS	Hospital transfer (Norwood op)		
21	24 + 1	MS, AA	Patent	TOP				
22	21 + 6	MS, AA, EFE	Patent	TOP				
23	21 + 6	MS, AA, EFE	Patent	TOP				
24	28 + 1	MS, AA, EFE	Patent	Lost to follow-up				
25	39 + 1	MS, AS	Patent	39 + 2	SVD	Hybrid proc. (PAB, PDA stenting)	BCS	
26	36 + 5	MS, AS, VSD	Patent	40 + 1	SVD	Hybrid proc. (PAB, PDA stenting)	CS II	
27	21 + 2	MS, AS, EFE	Patent	39 + 2	CS	Hybrid proc. (PAB, PDA stenting)	BCS	
28	19 + 6	MS, AS	Restrictive	38 + 1	CS	Hospital transfer		
29	31 + 6	BLV, VSD	Restrictive	35 + 3	CS	Compassionate care		
30	35 + 5	BLV	Patent	38 + 5	CS	No intervention necessary	SBF	
31	35 + 3	BLV, VSD	Patent	39 + 5	CS	No intervention necessary	SBF	
32	27 + 3	BLV, VSD	Patent	38 + 1	SVD	BCS	Ross	
33	34 + 1	BLV, VSD	Patent	40 + 0	CS	BCS		
34	21 + 0	BLV	Patent	36 + 0	CS	Hybrid proc. (PAB, PDA stenting)	BCS	
35	37 + 0	BLV	Restrictive	40 + 2	SVD	Hybrid proc. (PAB, PDA stenting)	BCS	
36	32 + 4	BLV, EFE	Patent	32 + 5	CS	Hybrid proc. (PAB, PDA stenting)	BCS	Ross
37	27 + 0	BLV, EFE	Patent	38 + 1	SVD	Hybrid proc. (PAB, PDA stenting)	CS II	TCPC
38	35 + 4	BLV, EFE	Patent	36 + 6	SVD	Hybrid proc. (PAB, PDA stenting)	CS II	TCPC
39	32 + 0	BLV, EFE	Restrictive	38 + 0	CS	Hospital transfer		
40	33 + 0	BLV	m.d	Lost to follow-up				
41	29 + 4	BLV, VSD	Patent	Lost to follow-up				

BLV, borderline left ventricle (small–diminutive left ventricle with intrinsically open valves (mitral stenosis and/or aortic stenosis); AA, aortic atresia; AS, aortic stenosis; CS, Cesarean section; EFE, endocardial fibroelastosis; MA, mitral atresia; m.d., missing data; MS, mitral stenosis; op, operation; proc., procedure; SVD, spontaneous vaginal delivery; VSD, ventricular septal defect; PAB, pulmonal arterial banding; PDA, persistent ductus arteriosus; TCPC, total cavopulmonary connection; CS II, Comprehensive Stage II; BCS, Biventricular correction surgery; HTX, orthotopic heart transplantation; SBF, sufficient biventricular function; Ross, Ross-OP; Ex, Exitus letalis

### Comparison of RV GLPSS and LSR between HLH and control fetuses

**Fig. 1:**
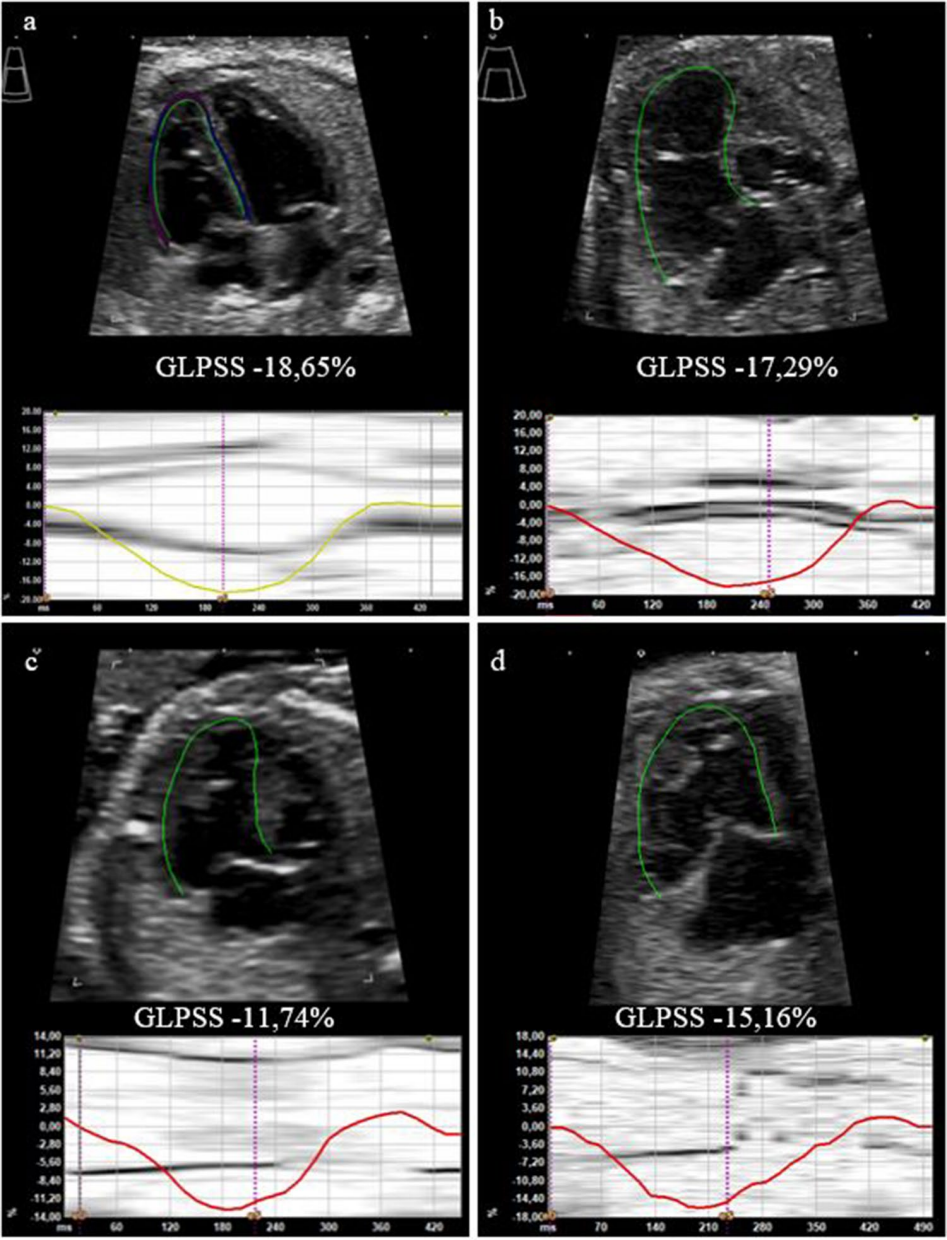
**a**, **b** Traced myocardial wall of the right ventricle (B-mode sonography) and global longitudinal strain (%) curves for one fetal heart cycle for a healthy fetus (**a**) and a fetus with hypoplastic left heart (HLH, MA, AA) at 22 + 1 weeks (**b**). **c**, **d** Traced myocardial wall of the right ventricle (B-mode-sonography) and global longitudinal strain (%) curves for one fetal heart cycle for a fetus with HLH (MS, AA) and EFE (**c**) at 21 + 6 weeks and for a fetus with HLH (MS, AS) without EFE (**d**) at 27 + 3 weeks. There is a tendency for lower GLPSS values in the case of HLH, especially for additionally existing EFE. *AA, aortic atresia; AS, aortic stenosis; EFE, endocardial fibroelastosis; MA, mitral atresia; MS, mitral stenosis

Relating to GLPSS values of RV, fetuses with HLH demonstrated lower mean values than control fetuses (− 15.65% ± 0.58 vs. − 16.80% ± 0.16, *p* = 0.065) without reaching statistical significance. Regarding RV LSR, fetuses with HLH showed slightly lower mean values than control fetuses (− 1.25 1/s ± 0.05 vs. − 1.29 1/s ± 0.03, *p* = 0.532). Figure 1 schematically shows lower RV GLPSS values in the case of an HLH (MA, AA) fetus compared to a healthy fetus.

### Comparison of RV GLPSS and LSR between HLH fetuses with and without LV-EFE

In the case of LV-EFE, analysis revealed statistically significant lower RV GLPSS mean values in comparison to such without LV-EFE (− 12.12% ± 1.02 vs. − 16.52% ± 0.60; *p* = 0.003; Table 2). Mean value of RV LSR was significantly lower in fetuses with LV-EFE than in those without additional LV-EFE (− 1.00 1/s ± 0.12 vs. − 1.32 1/s ± 0.05, *p* = 0.034; Table 2). Figure 1 schematically shows significantly lower RV GLPSS values in a case of an HLH fetus (MS, AA) with LV-EFE compared to a HLH fetus (MS, AS) without LV-EFE.

**Table 2:** Right ventricular global longitudinal peak systolic strain and right ventricular longitudinal strain rate for subgroup analysis hypoplastic left heart (HLH) with left ventricular endocardial fibroelastosis (LV-EFE) and HLH without LV-EFE

	HLH + LV-EFE (*n* = 11)	HLH w/o LV-EFE (*n* = 30)	Control group (*n* = 101)	*p*-value
RV GLPSS (%)	− 12.12 ± 1.02	− 16.52 ± 0.60	− 16.80 ± 0.16	0.002^a^
				0.003^b^
				0.649^c^
RV LSR (1/s)	− 1.00 ± 0.12	− 1.32 ± 0.05	− 1.29 ± 0.02	0.045^a^
				0.034^b^
				0.631^c^

Gestational age 27th week of pregnancy

*p*-values < 0.05 statistically significant

^a^HLH + LV-EFE versus control group

^b^HLH + LV-EFE versus HLH

^c^HLH versus control group

### Comparison of RV GLPSS and LSR between HLH fetuses with patent and restrictive FO

Subgroup analysis revealed lower RV GLPSS values in HLH fetuses with patent foramen ovale compared to those with restriction (− 15.33% ± 0.72 vs. − 16.64% ± 0.84; Table 3), without reaching statistical significance (*p* = 0.739). RV LSR values in HLH fetuses with patent foramen ovale were lower compared to HLH fetuses with restrictive foramen ovale without reaching statistical significance (− 1.22 1/s ± 0.06 vs. − 1.36 1/s ± 0.10, *p* = 0.811; Table 3).

**Table 3:** Right ventricular global longitudinal peak systolic strain and right ventricular longitudinal strain rate for subgroup analysis hypoplastic left heart (HLH) with patent foramen ovale and HLH with restrictive foramen ovale

	HLH	HLH	Control group (*n* = 101)	*p*-value
	FO patent (*n* = 31)	FO restrictive (*n* = 10)		
RV GLPSS (%)	− 15.33 ± 0.72	− 16.64 ± 0.84	− 16.80 ± 0.16	0.164^a^
				0.739^b^
				1.000^c^
RV LSR (1/s)	− 1.22 ± 0.06	− 1.36 ± 0.10	− 1.29 ± 0.03	0.825^a^
				0.811^b^
				1.000^c^

Gestational age 27th week of pregnancy

*p*-values < 0.05 statistically significant

^a^HLH FO patent versus control group

^b^HLH FO patent versus HLH FO restrictive

^c^HLH FO restrictive versus control group

### Comparison of RV GLPSS and LSR between HLH fetuses and control fetuses (excluding BLV cases)

Data analysis without BLV cases included 28 HLH fetuses. Relating to GLPSS values of RV, fetuses with HLH demonstrated lower mean values than control fetuses (− 15.55% ± 0.70 vs. − 16.85% ± 0.16, *p* = 0.08) without reaching statistical significance. Regarding RV LSR, fetuses with HLH showed slightly lower mean values than control fetuses (− 1.27 1/s ± 0.06 vs. − 1.29 1/s ± 0.02, *p* = 0.750). In the case of LV-EFE (*n* = 7), analysis revealed statistical significantly lower RV GLPSS mean values in comparison to such without LV-EFE (*n* = 21) (− 12.85% ± 1.42 vs. − 16.46% ± 0.72; *p* = 0.049; Table S1, supplementary material). Regarding RV LSR mean value was lower in fetuses with LV-EFE than in those without additional LV-EFE (− 1.18 1/s ± 0.15 vs. − 1.30 1/s ± 0.07; Table S1, supplementary material), without reaching statistical significance (*p* = 0.472). Significantly lower RV GLPSS values were found in HLH fetuses with patent FO (*n* = 21) compared to those with (*n* = 7).

FO restriction (− 14.88% ± 0.85 vs. − 17.52% ± 0.82, *p* = 0.038; Table S2, supplementary material). This is in contrast to the analysis with included BLV cases. Regarding RV LSR values in HLH fetuses with patent FO and restrictive FO no significant differences between groups could be observed (− 1.24 1/s ± 0.07 vs. − 1.37 1/s ± 0.12, *p* = 0.366; Table S2, supplementary material).

### Reproducibility of RV GLPSS and LSR

For the final analysis, 20 randomly selected fetuses were included. Interobserver as well as intraobserver variability revealed acceptable to moderate reproducibility of RV GLPSS and LSR values (Table 4). Inter- and intraobserver variability of 2D-STE indices is also illustrated by Bland–Altman plots in Fig. S1.

**Table 4:** Reproducibility analysis for right ventricular global longitudinal peak systolic strain and right ventricular longitudinal strain rate

	RV GLPSS (%)	RV LSR (1/s)
Observer 1	− 14.66 ± 3.47	− 1.19 ± 0.28
Observer 2	− 14.69 ± 2.98	− 1.16 ± 0.31
Interobserver ICC	0.943 [95% CI (0.862–0.977)]	0.739 [95% CI (0.452–0.887)]
Observer 1 1st	− 14.66 ± 3.47	− 1.19 ± 0.28
Observer 1 2nd	− 14.65 ± 3.71	− 1.22 ± 0.33
Intraobserver ICC	0.982 [95% CI (0.956–0.993)]	0.749 [95% CI (0.469–0.892)]

## Discussion

The aim of the present study was first, to evaluate RV systolic function in fetuses with HLH compared to healthy controls by 2D STE. We observed lower mean values for RV GLPSS in HLH fetuses suggesting an alteration of RV systolic function and contraction properties in fetal HLH. Second, we evaluated the possible influence of LV-EFE on RV function. Here, our results point towards a further deterioration in RV systolic function depending on the presence of LV-EFE. Third, we analyzed the impact of a restrictive FO on RV function. In HLH cases with restrictive FO we could not find any significant difference in RV deformation compared to cases with patent FO. However, after the exclusion of BLV cases, we observed statistically significant lower values for RV GLPSS in restricted FO cases. This suggests that the assumed functional impairment of RV counts more in cases that are definitely dependent on univentricular function.

In the case of HLH cardiac function decisively depends on RV performance. In the postnatal state, univentricular circulation is achieved by different surgical options with an RV acting as a systemic ventricle. Therefore, intrauterine changes in RV function could possibly have a high influence on the future single RV [9, 16, 17]. Furthermore, it is proven that postnatal RV dysfunction is rated as an important risk factor for the survival of HLH patients [30, 31].

For postnatal evaluation of RV systolic function, STE-based strain has been analyzed by Zaidi and colleagues. In their studies patients were divided into one group of HLH patients with normal RV function and one group with impaired function. Subsequently, RV GLPSS was calculated for both groups as well as for a healthy control group. RV GLPSS was not statistically different with a mean RV-GLPSS of − 20.5 ± 3.6% in the normal group versus − 17.9 ± 2.6% in the HLH group with preserved function. However, the investigation leads to worse parameters in the case of HLH patients with poor function. We found this to be quite impressive. RV GLPSS in the HLH group with poor RV function only reached − 12.1 ± 4.0%. Moreover, Zaidi et al. figured out that RV GLPSS could differentiate between preserved and reduced function. Therefore, they adopted a cut-off value of − 16% [32]. This is in line with our prenatal findings, as HLH fetuses showed a mean RV GLPSS value < 16% (− 15.65%) compared to controls (− 16.80%). Especially in the case of concomitant EFE, which is presumed to deteriorate RV function, RV GLPSS value (− 12.12%) is far beyond the cut-off value of − 16%. These findings may indicate the predictive value of prenatal RV function assessment regarding the poor functional status of the single RV in the future. Therefore, for treatment planning, it seems to be important to find parameters for identifying systolic dysfunction and therefore risk factors for higher mortality of HLH patients. Miller et al. assessed prenatal altered RV performance as a probable predictive parameter for postnatal RV dysfunction. In their study using velocity vector imaging they described that RV GLPSS was significantly lower in patients with HLH compared to healthy controls before other hemodynamic changes could have influenced the future systemic RV. Consequently, this is proof for systolic dysfunction, which—in combination with the described diastolic dysfunction—could be considered as an origin of the decreased output in HLH fetuses [33]. Former studies, of our group using tissue Doppler imaging techniques [9, 14] revealed a primarily diastolic dysfunction of the RV in fetal HLH consistent with other research groups [16]. In addition, Natarajan et al. realized a subgroup analysis with regard to LV-EFE that shows the most striking differences in RV mechanics in patients with prevailing LV-EFE. This is in line with our own results regarding RV function in HLH fetuses with and without LV-EFE measured by M-Mode, pulsed wave Doppler and tissue Doppler imaging techniques [8]. This study on 2D STE in HLH also underscores the influence of the presence of LV-EFE even on RV systolic function.

The course of postnatal treatment showed, that in two BLV cases no intervention was necessary and six BLV cases had a biventricular repair. It seems to be difficult to assess the degree of ventricular development prenatally and to determine whether a biventricular or a univentricular repair is necessary, which makes the inclusion of BLV cases questionable. As mentioned by Kaplinski and Cohen there are new treatment strategies for BLV but the assessment of the severity of left ventricle hypoplasia remains challenging [34]. This is due to morphological and physiological changes happening during fetal life. Studies about the prenatal evaluation of myocardial function in BLV cases appear to be rare, which supports the suggestion for further studies to exclude those patients and maybe investigate this problem in their own study.

There were several limitations to our study. On the one hand, the acquisition of a proper apical or basal four-chamber view is complicated by fetal mobility, respiratory movement and by maternal characteristics like body mass index.

On the other hand, the relatively small sample sizes increase the risk of type 2 statistical error. Especially after the exclusion of BLV cases, one has to keep in mind, that even smaller study population and subgroups could influence the general informative value of this study. Another disadvantage is the retrospective design of this study which entails a lack of influence regarding the type, quality and completeness of enrolled data.

The control group is of lower gestational age with 26.0 weeks versus 29.1 in the HLH group. As longitudinal strain is decreasing with gestational age this may explain, at least in part, a higher RV GLPSS in the control group. Furthermore, there is a lack of prenatal fetal follow-up and information on valve regurgitation that may affect RV systolic function interpretation is not provided.

Furthermore, in our study only longitudinal deformation was examined. We did not examine circumferential, radial and rotational deformation. However, myocardial contraction is three-dimensional and it would of course be interesting to investigate myocardial thickening and twisting as well. Therefore, it is questionable whether the assessment of longitudinal strain alone adequately describes RV function. A normal myocardial contraction and relaxation depending on the rearrangement of its microstructures [35]. Ma et al. recently described the altered myocardial fiber trajectories in the RV with diffusion tensor imaging in post-mortem HLH fetuses. Using different parameters for alignment and microstructure, they found morphological and functional changes in the RV in HLHS fetuses. Furthermore, cardiac function was related to the orientation patterns of myocardial fibers. The RV myocardium in HLH showed a more compact and organized adaptation, more resembling the global myocardial helix [36]. With RV volume loading in HLH, RV sphericity increases. The increasing sphericity alone will affect the axis of motion of the HLH RV compared with a “normal” RV and could also artificially condition reduced shortening. Out-of-plane motion could potentially impact the TomTec tracking algorithm.

Reduced RV global longitudinal strain values in fetuses with HLH (and LV-EFE) point towards antenatal changes in myocardial function thus questioning the concept of altered postnatal RV function being the consequence of long-term exposure to increased pressure and volume load on LV only. We hope that further explorations by STE technique can expand knowledge about RV contraction abilities in HLH fetuses with possible influence on perinatal outcome.

## Supplementary Information

Below is the link to the electronic supplementary material.

**Fig. S1**: Graphical representation of the Bland–Altman-analysis: plotted mean values of the measured value pairs against their differences. Interobserverreliability (**a**, **b**) and Intraobserverreliability (**c**, **d**) for RV GLPSS and RV LSR. (TIFF 3856 kb)

**Table S1**: Right ventricular global longitudinal peak systolic strain and right ventricular longitudinal strain rate for subgroup analysis HLH with LV-EFE and HLH without LV-EFE. Analysis without Borderline LV cases. (DOCX 17 kb)

**Table S2**: Right ventricular global longitudinal peak systolic strain and right ventricular longitudinal strain rate for subgroup analysis HLH with patent foramen ovale and HLH with restrictive foramen ovale. Analysis without Borderline LV cases. (DOCX 17 kb)
