# Supplementary material for: Evaluation of right ventricular myocardial deformation properties in fetal hypoplastic left heart by two-dimensional speckle tracking echocardiography
Source: Arch Gynecol Obstet. 2023 Feb 10;307(3):699–708. doi: 10.1007/s00404-022-06857-x (PMC9984504; doi:10.1007/s00404-022-06857-x)
Supplement: Supplementary file 2 — Table S1: Right ventricular global longitudinal peak systolic strain and right ventricular longitudinal strain rate for subgroup analysis HLH with LV-EFE and HLH without LV-EFE. Analysis without Borderline LV cases. (DOCX 17 kb) [file 404_2022_6857_MOESM2_ESM.docx]

**Tab. S1:**

Right ventricular global longitudinal peak systolic strain and right ventricular longitudinal strain rate for subgroup analysis HLH with LV-EFE and HLH without LV-EFE. Analysis without Borderline LV cases.

|  | **HLH + LV-EFE**  **(n=7)** | **HLH w/o LV-EFE**  **(n=21)** | **Control group**  **(n=101)** | **p-value** |
| --- | --- | --- | --- | --- |
| RV GLPSS (%) | -12.85 ± 1.42 | -16.46 ± 0.72 | -16.85 ± 0.16 | 0.030^1^  0.049^2^  0.606^3^ |
| RV LSR (1/s) | -1.18 ± 0.15 | -1.30 ± 0.07 | -1.29 ± 0.02 | 0.484^1^  0.472^2^  0.879^3^ |

Gestational age 27th week of pregnancy

^1^HLH + LV-EFE vs. control group

^2^HLH + LV-EFE vs. HLH w/o LV-EFE

^3^HLH w/o LV-EFE vs. control group

p-values < 0.05 statistically significant
