# Supplementary material for: Evaluation of right ventricular myocardial deformation properties in fetal hypoplastic left heart by two-dimensional speckle tracking echocardiography
Source: Arch Gynecol Obstet. 2023 Feb 10;307(3):699–708. doi: 10.1007/s00404-022-06857-x (PMC9984504; doi:10.1007/s00404-022-06857-x)
Supplement: Supplementary file 3 — Table S2: Right ventricular global longitudinal peak systolic strain and right ventricular longitudinal strain rate for subgroup analysis HLH with patent foramen ovale and HLH with restrictive foramen ovale. Analysis without Borderline LV cases. (DOCX 17 kb) [file 404_2022_6857_MOESM3_ESM.docx]

**Tab. S2:**

Right ventricular global longitudinal peak systolic strain and right ventricular longitudinal strain rate for subgroup analysis HLH with patent foramen ovale and HLH with restrictive foramen ovale. Analysis without Borderline LV cases.

|  | **HLH**  **FO patent**  **(n=21)** | **HLH**  **FO restrictive**  **(n=7)** | **Control group**  **(n=101)** | **p-value** |
| --- | --- | --- | --- | --- |
| RV GLPSS (%) | -14.88 ± 0.85 | -17.52 ± 0.82 | -16.85 ± 0.16 | 0.033^1^  0.038^2^  0.454^3^ |
| RV LSR (1/s) | -1.24 ± 0.07 | -1.37 ± 0.12 | -1.29 ± 0.02 | 0.473^1^  0.366^2^  0.550^3^ |

Gestational age 27th week of pregnancy

^1^HLH FO patent vs. control group

^2^HLH FO patent vs. HLH FO restrictive

^3^HLH FO restrictive vs. control group

p-values < 0.05 statistically significant
